# PET and MRI for the evaluation of regional myocardial perfusion and wall thickening after myocardial infarction

**DOI:** 10.1007/s00259-012-2085-0

**Published:** 2012-03-13

**Authors:** Riemer H. J. A. Slart, Julius Glauche, Reza Golestani, Clark J. Zeebregts, Jan W. Jansen, Rudi A. J. O. Dierckx, Matthijs Oudkerk, Tineke P. Willems, Andor W. J. M. Glaudemans, Hendrikus H. Boersma, René A. Tio

**Affiliations:** 1 Department of Nuclear Medicine and Molecular Imaging, University Medical Center Groningen, University of Groningen, Hanzeplein 1, P.O. Box 30001, 9700 RB Groningen, The Netherlands; 2 Department of Cardiology, University Medical Center Groningen, University of Groningen, Groningen, The Netherlands; 3 Department of Surgery, Division of Vascular Surgery, University Medical Center Groningen, University of Groningen, Groningen, The Netherlands; 4 Department of Radiology, University Medical Center Groningen, University of Groningen, Groningen, The Netherlands; 5 Clinical and Hospital Pharmacy, University Medical Center Groningen, University of Groningen, Groningen, The Netherlands; 6 Department of Nuclear Medicine, Ghent University Hospital, Ghent, Belgium

**Keywords:** Myocardial infarction, Left ventricular function, Myocardial perfusion imaging, Cardiac MRI

## Abstract

**Background:**

Deterioration of left ventricular (LV) function after myocardial infarction (MI) is a major cause of heart failure. Myocardial perfusion performance may play an important role in deterioration or improvement in LV function after MI. The aim of this study was to evaluate the myocardial perfusion reserve (MPR) and stress perfusion in deteriorating and non-deteriorating LV segments in patients after MI by PET and MRI, respectively.

**Methods:**

Regional wall thickening of 352 segments in 22 patients was assessed at 4 and 24 months after MI by cardiac MRI. PET was performed to evaluate MPR and adenosine stress ^13^N-ammonia perfusion 24 months after MI. Segments were divided into four groups according to deterioration or improvement in wall thickening.

**Results:**

Normal functional segments at 4 months after MI that remained stable had a significantly higher mean MPR and mean stress perfusion PET value than deteriorated segments (*p* < 0.001). Furthermore, dysfunctional segments that improved had a significantly higher mean stress perfusion PET value than dysfunctional segments that remained dysfunctional (*p* < 0.001).

**Conclusion:**

This study demonstrated the additional value of myocardial perfusion assessment in relation to the functional integrity of the injured myocardium. Segmental functional LV improvement after MI was associated with better regional myocardial perfusion characteristics. Furthermore, the amount of wall thickening reduction was associated with regional myocardial perfusion abnormalities in patients after MI.

## Introduction

During recent decades, substantial improvements in the treatment strategies in patients with myocardial infarction (MI) have been achieved. Although this has greatly reduced the rate of early mortality, MI-induced heart failure remains a major cause of MI-associated morbidity and late mortality [1]. After MI, spared myocardium should compensate for tissue fibrosis due to MI. Two important determinants of functional outcome are the amount of remaining myocardial tissue and the myocardial perfusion dynamics of the spared myocardium.

Myocardial perfusion performance depends on the condition of large (epicardial) and small (resistance) vessels. Deficient myocardial perfusion is considered to play a key role in pathophysiological processes involved in MI-induced heart failure [2]. Inadequate angiogenesis, microvascular and cardiomyocyte dysfunction, scar formation, and insufficient perfusion via the collateral coronary arteries will lead to deterioration of heart function [3]. On the other hand, the increase in myocardial perfusion caused by angiogenesis and a sufficient number of collateral arteries may result in improvement in myocardial pump function. Angiogenesis can be stimulated by means of gene therapy, e.g. VEGF [4, 5], or experimentally by stem cell therapy [6].

PET-based myocardial perfusion imaging is used for absolute quantification of myocardial perfusion (millilitres per minute per gram) at the global and regional levels [5]. Due to accuracy of the myocardial perfusion imaging, myocardial perfusion reserve (MPR, the ratio of near-maximal myocardial blood flow to the baseline value) can be calculated. MPR and stress perfusion PET are used to assess epicardial coronary arteries as well as microcirculatory function and the myocardial hemodynamic reserve [7, 8]. MRI is a noninvasive imaging modality which provides clinicians with accurate information of global and regional heart function with high spatial and temporal resolution [2, 6]. MR images allow delineation of the myocardium, thus allowing measurements of systolic wall thickening (WT). Combined application of both PET and MRI will provide clinicians with additional information on perfusion and function parameters of the myocardium. Based on the relationship between myocardial perfusion and myocardial function as previously described in patients with idiopathic dilated cardiomyopathy [9–11], we hypothesized that myocardial perfusion is associated with changes in regional myocardial function after MI. In this study, we therefore explored the relationship between PET MPR, adenosine stress ^13^N-ammonia perfusion, and changes in regional myocardial WT after MI as assessed with MRI.

## Materials and methods

### Patients

Patients with MI admitted to the University Medical Center Groningen (UMCG) were retrospectively included in this study. They all underwent percutaneous coronary intervention (PCI). Before starting the PCI procedure patients were treated with aspirin, heparin and clopidogrel. After PCI patients received a beta-blocker, aspirin, lipid-lowering therapy, and a renin-angiotensin-aldosterone system inhibitor combined with clopidogrel. Patients participated in the HEBE trial [6] and underwent a PET scan and were included by random sampling for infarct size determination and detection of ischaemia. No differences in regional or global LV function were observed between the HEBE treatment groups; therefore we analysed our sample as a whole.

### MRI

At 4 and 24 months after infarction cardiac MRI was performed to assess left ventricular (LV) function. A 1.5-T MRI scanner with a four-element phased array cardiac receiver coil was used. Images were analysed using a dedicated software package (QMass 7.2; Medis, Leiden, The Netherlands). Endocardial and epicardial borders in end-diastolic and end-systolic images were outlined manually on short-axis slices, covering the entire LV from base to apex including the papillary muscles to the volume. For regional analysis the LV was divided into 16 different segments. Segmental end-diastolic wall thickness (EDWT) and end-systolic wall thickness (ESWT) were determined in millimetres. WT was calculated in each segment by subtracting EDWT from ESWT. At each time-point a cut-off value of 3 mm was applied to consider segments as functional versus dysfunctional [12]. All segments were divided into four groups based on functionality at two time-points (Table 1).

**Table 1:** Myocardial segment groups in relation to WT at 4 and 24 months after MI. WT <3 mm was considered as dysfunctional and WT ≥3 mm was considered as normal

Group	Segments at 4 months	Segments at 24 months
1	Normal	Normal
2	Normal	Dysfunctional
3	Dysfunctional	Normal
4	Dysfunctional	Dysfunctional

### PET

The patients underwent a rest ^13^N-ammonia PET scan and an adenosine-induced stress ^13^N-ammonia PET scan 24 months after MI. Patients refrained from caffeinated beverages from 12 h prior to scanning. Imaging was performed with the patient supine on an ECAT HR+ PET camera (Siemens Medical Systems, Knoxville, TN). A transmission scan for further attenuation correction was acquired for 5 min using an external ring source filled with ^68^Ge/^68^Ga. Accidental coincidence and dead time were automatically corrected. Myocardial perfusion imaging at rest was started 2 min after injection of 400 MBq of ^13^N-ammonia and was continued for 15 min. The same protocol was used 6 min after intravenous injection of 0.14 mg/kg per minute adenosine to acquire stress myocardial perfusion images. A parametric polar map was generated from the PET data. These data were corrected for partial volume effects and activity spillover from the blood pool. Regional (stress and rest) myocardial blood flow was calculated using a 17-segment model, but reformatted in a 16 segment model corresponding to the obtained MRI data. Segmental MPR was calculated by dividing the adenosine stress ^13^N-ammonia PET result by the rate pressure product normalized rest ^13^N-ammonia for each segment.

### Data analysis

The results are expressed as means ± SD. The Kolmogorov-Smirnov test was used to assess whether the MPR and WT values were distributed normally. Due to a non-normal distribution of WT values in the sample, the Kruskal-Wallis test was used to compare the MPR and stress perfusion PET values between different groups of WT. A paired Student’s *t*-test was used to assess differences in WT between baseline and follow-up MRI. The significance level was set at 0.05.

## Results

We evaluated 352 segments in 22 patients. There were no revascularization procedures between the first and second time-points. Descriptive data on cardiovascular risk factors, LV ejection fraction (LVEF), MPR, wall thickness and WT at both time-points are shown in Table 2.

**Table 2:** Demographic and clinical characteristics of the 22 patients

Variable	
Age (years, mean ± SD)	61.7 ± 6.8
Female (%)	13.6
Family history of cardiovascular diseases (%)	40.9
Smoker (%)	31.8
Diabetes mellitus (%)	4.5
Hypertension (%)	36.4
Hypercholesterolaemia (%)	27.3
LVEF (%, mean ± SD)	
4 months	38.6 ± 7.5
24 months	41.2 ± 8.9
MPR (mean ± SD)	1.5 ± 0.6
Stress ^13^N-ammonia (ml/min/100 g, mean ± SD)	107.2 ± 46.7
WT (mean ± SD)	
4 months (mm)	3.3 ± 2.2
24 months (mm)	3.8 ± 2.6
ΔWT (mm, mean ± SD)^a^	0.5 ± 1.8

^a^Difference in WT between 4 and 24 months.

The first MRI scan was performed 4 months after MI. In all 352 segments the mean values of EDWT, ESWT and WT were 7.0 ± 1.8, 10.4 ± 2.9, and 3.5 ± 2.1 mm, respectively. The second MRI scan was performed 24 months after MI to follow-up the patients’ myocardial function. The mean values of EDWT, ESWT and WT in the second scan were 7.3 ± 2.2 mm (*p* < 0.01), 11.4 ± 3.5 mm (*p* < 0.01) and 4.0 ± 2.5 mm (*p* < 0.01), respectively.

The segments were divided according to the change in WT functionality during the 20-month period between the two MRI scans. At 24 months after MI, myocardial perfusion as assessed by ^13^N-ammonia PET and MPR was calculated. Table 3 shows the observed MPR, myocardial perfusion values, and WT in the different groups. The group 1 pattern (normal segments at both time-points) was seen in 183 segments (WT 5.0 ± 1.4 mm and 5.6 ± 1.7 mm at 4 and 24 months, respectively) in 21 patients. The group 2 pattern (normal segments at 4 months but WT below the normal limit at 24 months) was seen in 28 segments (WT 3.8 ± 0.7 mm and 1.8 ± 0.9 mm at 4 and 24 months, respectively) in 16 patients. The group 3 pattern (dysfunctional segments at 4 months but WT improved at 24 months) was seen in 51 segments (WT 2.0 ± 0.8 mm at 4 months and 4.5 ± 1.3 mm at 24 months) in 19 patients. The group 4 pattern (dysfunctional segments at both time-points) was seen in 90 segments (WT 1.2 ± 1.1 mm and 1.2 ± 1.7 mm at 4 and 24 months, respectively) in 19 patients.

**Table 3:** Regional characteristics in relation to WT status. The data are presented as means ± SD (for the definitions of the segment groups, see Table 1)

Group	No. of segments	MPR	Myocardial perfusion (ml/min/100 g)		WT (mm)	
			Stress PET	Rest PET	4 months	24 months
1	183	1.7 ± 0.6 (0.1–3.7)	128.2 ± 48.3 (5–347)	79.3 ± 20.6 (34.3–140)	5.0 ± 1.4 (3.0–10.5)	5.6 ± 1.7 (2.7–11.4)
2	28	1.2 ± 0.5 (0.1–2.3)	82. 7 ± 30.2 (9–157)	71.2 ± 16.9 (31.4–105.3)	3.8 ± 0.7 (3.0–5.7)	1.8 ± 0.9 (−0.8–2.9)
3	51	1.5 ± 0.6 (0.1–3.4)	100.7 ± 36.2 (6–170)	70.8 ± 21.5 (33.8–118.1)	2.0 ± 0.8 (0.3–2.9)	4.5 ± 1.3 (3.0–7.7)
4	90	1.3 ± 0.5 (0.2–3.5)	83.2 ± 34.7 (20–198)	65.8 ± 21.4 (26.2–137.1)	1.2 ± 1.1 (−1.5–2.8)	1.2 ± 1.7 (−2.4–2.9)

The mean MPR values were 1.7 ± 0.6, 1.2 ± 0.5, 1.5 ± 0.6, and 1.3 ± 0.5 in groups 1–4, respectively. Overall, the mean MPR in the 352 segments was 1.5 ± 0.6 (Table 3). The mean stress perfusion PET values in groups 1–4 were 128.2 ± 48.3, 82.7 ± 30.2, 100.7 ± 36.2 and 83.2 ± 34.7 ml/min/100 g, respectively. Groups 1 and 2 (both normal functional segments at 4 months) showed significantly lower mean MPR and stress perfusion PET values in the deteriorated segments at 24 months (perfusion 128.2 ± 48.3 vs. 82. 7 ± 30.2 ml/min/100 g; *p* < 0.001) than the group with remaining normal functional segments. Groups 3 and 4 (both dysfunctional segments at 4 months) showed higher mean MPR and stress perfusion PET values in the functionally improved segments at 24 months (perfusion 100.7 ± 36.2 vs. 83.2 ± 34.7 mL/min/100 g, *p* = 0.001; MPR, borderline significance, *p* = 0.05) than the group with remaining dysfunctional segments (Table 3).

## Discussion

In the present study we evaluated the relationship between changes in regional myocardial WT between 4 and 24 months after MI and PET myocardial perfusion. The main finding of this study was the association between segmental LV function deterioration and a lower stress perfusion as well as perfusion reserve.

Congestive heart failure is the leading cause of MI-associated mortality and mortality [1]. The influence of myocardial perfusion and microvascular condition on myocardial contractility has been evaluated in several studies in patients with nonischaemic heart failure [11, 13, 14]. Myocardial remodelling in response to functional loss after an MI greatly depends on sufficient perfusion and oxygen supply. Reduced MPR and stress perfusion PET values as were found in the deteriorated segments may imply insufficient oxygen supply as a cause of deterioration. Improvement in segmental function may on the one hand relate to recovery after myocardial stunning and on the other hand reflect compensation by the healthy/spared myocardium for the loss of infarcted tissue. Deterioration of previously functional segments may reflect the inability of the tissue to adapt to the increase in metabolism needed for this compensation. The present study, however, could not differentiate between cause and consequence. The results of this study may merely reflect the difference in myocardial perfusion between normal and remodelled myocardium.

The prognostic role of regional and global MPR in patients with hypertrophic cardiomyopathy [13, 15], idiopathic LV dysfunction [16] and ischaemic heart disease [17, 18] has been shown in previous studies. The present study did not evaluate the prognostic value of myocardial perfusion measurement but focused on the possible mechanism of deteriorating LV function after MI. These data do, however, further elaborate on MPR and stress perfusion PET values in a well-characterized group of patients with a previous MI. The observation that contractile deterioration is directly associated with reduced perfusion dynamics further underlines the microcirculation as an important diagnostic and possible therapeutic target in patients following MI. Death of cardiomyocytes is followed by inflammation, proliferating myofibroblasts and migration of endothelial cells into the infarct zone replacing dead tissue with granulation tissue [19]. Concomitantly, vβ3 integrins are upregulated in the infarct region [20]. It is well recognized that the expression of the b3 integrins contributes to angiogenesis in the peri-infarct zone as a part of the remodelling process, and occurs early, peaking about 7 days after MI. It has been postulated that the extent of neovascularization determines the functional integrity of the (spared) myocardium [2].

Using serial MRI measurements after MI functional segmental LV changes can be identified. At present the clinical importance of such changes remains to be determined. PET perfusion imaging may be of additional clinical assistance. It would be worth evaluating the usefulness of prospective PET perfusion assessment in the prediction of LV deterioration/heart failure in later stages. If such an approach was proved to be of value, it could be used for guiding personalized therapy in patients with heart disease.

A limitation of this study was the relatively low number of included patients, but the number of analysed LV segments was large. Also data on long-term outcomes were not available in this study, but need to be collected in future studies. These data are of importance for determining if stress perfusion and MPR PET are still linked over the long term with LV the contractility pattern after MI.

In conclusion, the combination of PET and MRI is a powerful tool for evaluating the relationship between myocardial perfusion and contractility changes after MI at a regional level. The observation that functional deterioration is accompanied by reduced stress perfusion as well as perfusion reserve further emphasizes the relationship between sufficient oxygen supply and cardiac remodelling after MI. Future research should establish whether prognostic evaluation of perfusion reserve could identify patients at risk of developing heart failure.
